# Oral health-related quality of life in children and adolescents with osteogenesis imperfecta: cross-sectional study

**DOI:** 10.1186/s13023-018-0935-y

**Published:** 2018-10-25

**Authors:** Mohammadamin Najirad, Mang Shin Ma, Frank Rauch, Vernon Reid Sutton, Brendan Lee, Jean-Marc Retrouvey, Members of the BBD, Sandesh C. S. Nagamani, Francis Glorieux, Paul Esposito, Eric Rush, Michael Bober, David Eyre, Danielle Gomez, Gerald Harris, Tracy Hart, Mahim Jain, Deborah Krakow, Jeffrey Krischer, Eric Orwoll, Cathleen Raggio, Peter Smith, Laura Tosi, Shahrokh Esfandiari

**Affiliations:** 1 Division of Oral Health and Society, Faculty of Dentistry, McGill University, 2001 McGill College, Suite 500, Montreal, Quebec H3A 1G1 Canada; 2 Shriners Hospital for Children, Mount Royal, 1529 Cedar Avenue, Montreal, Quebec H3G 1A6 Canada; 3 Molecular and Human Genetics, Baylor College of Medicine, 6701 Fannin CC1560, Houston, TX 77030 USA; 4 Department of Orthodontics, Faculty of Dentistry, McGill University, 2001 McGill College, Suite 500, Montreal, Quebec H3A 1G1 Canada

**Keywords:** Osteogenesis imperfecta, Oral health-related quality of life, Child perceptions questionnaires

## Abstract

**Background:**

Osteogenesis imperfecta (OI) affects dental and craniofacial development and may therefore impair Oral Health-Related Quality of Life (OHRQoL). However, little is known about OHRQoL in children and adolescents with OI. The aim of this study was to explore the influence of OI severity on oral health-related quality of life in children and adolescents.

**Methods:**

Children and adolescents aged 8–14 years were recruited in the context of a multicenter longitudinal study (Brittle Bone Disease Consortium) that enrolls individuals with OI in 10 centers across North America. OHRQoL was assessed using the Child Perceptions Questionnaire (CPQ) versions for 8 to 10-year-olds (CPQ_8–10_) and for 11 to 14-year-olds (CPQ_11–14_).

**Results:**

A total of 138 children and adolescents (62% girls) diagnosed with OI types I, III, IV, V and VI (*n* = 65, 30, 37, 4 and 2, respectively) participated in the study. CPQ_8–10_ scores were similar between OI types in children aged 8 to 10 years. In the 11 to 14-year-old group, CPQ_11–14_-scores were significantly higher (i.e. worse) for OI types III (24.7 [SD 12.5]) and IV (23.1 [SD 14.8]) than for OI type I (16.5 [SD 12.8]) (*P* < 0.05). The difference between OI types was due to the association between OI types and the functional limitations domain, as OI types III and IV were associated with significantly higher grade of functional limitations compared to OI type I.

**Conclusion:**

The severity of OI impacts OHRQoL in adolescents aged 11 to 14 years, but not in children age 8 to 10 years.

**Electronic supplementary material:**

The online version of this article (10.1186/s13023-018-0935-y) contains supplementary material, which is available to authorized users.

## Background

Osteogenesis Imperfecta (OI), also known as “brittle bone disease,” is a rare heritable disorder (prevalence 8 per 100,000 people) that is characterized by recurrent fractures and, in severe cases, skeletal deformities [1]. Extra-skeletal features such as blue or grey discoloration of sclera and discoloration and brittleness of teeth (dentinogenesis imperfecta, DI) can be associated. In about 90% of individuals with a clinical diagnosis of OI, a dominant mutation in the genes that code for type 1 collagen alpha chains (*COL1A1* and *COL1A2*) can be identified as the cause of the disease [2]. OI has traditionally been classified into four clinical types reflecting the severity of the phenotype (type I – mild; type II - neonatal lethal; type III – severe; type IV - moderately severe). The number of OI types has subsequently been expanded based on distinct clinical features and later based on the underlying genetic etiology [1, 3]. There is presently no cure for the disease, but pharmacological therapies using bisphosphonate drugs are widely used to strengthen bones, decrease the pain and fracture rates [3].

The World Health Organization (WHO) defines quality of life (QoL) as the “individual’s perception of their position in life in the context of the culture and value systems in which they live and in relation to their goals, expectations, standards, and concerns” [4]. The term “Health-Related Quality of Life” (HRQoL) narrows QoL to aspects relevant to health [5], and the term “oral health-related quality of life” (OHRQoL) focuses on physical, psychological, and social impacts of oral and orofacial conditions and disparities in oral health on overall health and QoL of individuals.

The pathological effects of OI on dental tissues and oral cavity usually develop in early life and may therefore influence OHRQoL during childhood and adolescence. Orofacial manifestations often associated with OI include DI, posterior open bite (lateral open bite), class III dental and skeletal malocclusion, anterior and posterior crossbites and impacted teeth [6]. The degree of the oral manifestations seem to be most severe in OI type III, as this type is associated with more severe craniofacial deformities and a higher prevalence of DI than milder forms of OI, such as OI type I and IV [7, 8].

Previous studies assessing HRQoL in OI patients has shown that children and adolescents with OI reported scores equivalent to the healthy population except in the physical domain (functional constraint) where they have presented significant lower scores [9]. Furthermore, children and adolescents with OI type III and IV have lower physical HRQoL compared to OI type I while sharing same range scores in other domains (emotional, school, and social) [10]. Until now little is known about OHRQoL in children with OI. It is unclear whether OHRQoL differs between OI types and between children and teenagers with OI.

In the present study we therefore assessed OHRQoL in children and adolescents with OI who participated in a multicenter study exploring the natural history of the disease. We hypothesized that the more severe phenotype in OI type III would result in lower OHRQoL compared to less severe OI types.

## Methods

### Study participants, recruitment, and setting

Study participants were recruited through the Brittle Bone Disease Consortium [11] that comprises several specialized centers from across North America (Houston, Montreal, Chicago, Baltimore, Portland, Washington DC, New York, Omaha, Los Angeles, Tampa). The consortium is a Rare Disease Clinical Research Network that is funded by the National Institutes of Health. One of the projects conducted by the consortium is a natural history study. The study was approved at all participating study centers, and all study participants or their legal guardians provided informed consent.

The present evaluation includes all children and adolescents with any OI type for whom OHRQoL data could be obtained in the first two study years from 6th of August 2015 to 3rd of August 2017. As the two pediatric OHRQoL instruments used in the study were specific for the age ranges from 8 to 10 years and from 11 to 14 years, respectively, the present analysis includes children and adolescents from 8 to 14 years of age. OHRQoL questionnaires were collected on paper at participating study sites and entered into an online data capture system that is maintained by the study Data Management and Coordinating Center (University of South Florida).

### Data collection

OHRQoL was evaluated using the Child Perception Questionnaire (CPQ). The CPQ_8–10_ containing 25 questions was used for children between 8 and 10 years of age (see Additional file 1) [12], the CPQ_11–14_ comprising 37 questions was used for individuals aged 11 to 14 years (see Additional File 2) [13]. Study participants were asked to complete the questionnaire unassisted by parents or investigators [14, 15]. These instruments are comprised of four health domains: oral symptoms, functional limitation, emotional well-being and social well-being related to oral health conditions. All questions consider the frequency of events in relation to the condition of the mouth or teeth over the previous four weeks (CPQ_8–10_) or three months (CPQ_11–14_). The responses to questions are scored on a frequency scale using the following response options and associated codes: ‘Never = 0’; ‘Once/twice = 1’; ‘Sometimes = 2’; Ofte*n* = 3′, and ‘Everyday/Almost every day = 4’. The questionnaires also contain two single-item global ratings. Additive subscale CPQ scores (domain-specific score) are computed by summing response codes. The overall CPQ scores are computed by adding up all four domain subscale scores, which may range from 0 to 100 for CPQ_8–10_ and 0 to 148 for CPQ_11–14_. Higher scores denote worse OHRQoL [12–14, 16]. The validity, reliability, and responsiveness of this measure have been established in various settings [17–22].

### Data analysis

Statistical analyses were performed using Stata 13.0 software (StataCorp. 2013. *Stata Statistical Software: Release 13*. College Station, TX: StataCorp LP) with a 5% significance level. Collected variables were classified into three levels: (1) Sociodemographic characteristics; (2) medical and physical conditions; and (3) OHRQoL. Missing values for some CPQ constituent questions (6% of questionnaire data fields) and were substituted with the mean value of that variable across each OI type (single imputation method). Descriptive and univariate analyses were performed across different types of OI separately for each age group. Welch’s t-test (independent samples t-test) was employed to handle the unequal variances and sample sizes between the groups of binary variables. When the sample size of a group was less than 15 patients, the Mann-Whitney U-test (non-parametric) was performed to assess for the significance of differences between two groups. To determine the significant relationship between categorical variables, Chi-square test or Fisher’s exact test for contingency tables with small cell counts were employed. CPQ scores and their constituent subscale scores were transformed to ordinal variables using their 33rd and 66th percentiles. Age, gender, race, and having a family history were identified as the minimum set of potential confounders, to be included in the multivariate analyses. Multivariable ordinal logistic regression analyses were employed to estimate the total effect of OI types on CPQ score and its constituent domains.

## Results

A total of 138 individuals (62% females) aged 8–14 years (11.6 ± 2.1 years) and diagnosed with OI types I, III, IV, V and VI (*n* = 65, 30, 34, 6 and 3, respectively) participated in the study. As the number of participants with OI types V and VI was too small for meaningful statistical analysis, these two OI types were analyzed in one group (Others) (Table 1). All patients were living with their biological parents. Six participants (OI type I, *n* = 3; OI type III, *n* = 1; OI type IV, *n* = 2) were homeschooled, the others attended school.

**Table 1:** Characteristics of the 8 to 10-year-old group

Patients aged between 8 and 10	OI I	OI III	OI IV	Others	All
Sociodemographic Characteristics					
Enrolment number – *n* (%)	26 (46)	16 (29)	11 (20)	3 (5)	56 (100)
Female	13 (50)	12 (75)	7 (64)	2 (66)	34 (61)
Age – mean (SD)	9.3 (1.0)	9.2 (0.9)	9.8 (0.5)	9.6 (0.5)	9.4 (0.9)
Race (White) – *n* (%)	22 (85)	13 (81)	5 (45) ^**c**^	3 (100)	43 (77)
others	4 (15)	3 (19)	6 (55) ^**c**^	0 (0)	13 (23)
Insurance status (Private) – *n* (%)	20 (77)	8 (50)	8 (73)	2 (67)	38 (68)
Medicare/Medicaid	6 (23)	8 (50)	3 (27)	1 (33)	18 (32)
Pertinent Medical and Physical Conditions					
Family history (Yes) – *n* (%)	19 (73) ^**a**^	2 (12)	3 (21) ^**c**^	3 (100)	27 (48)
Chronic pain in body (Yes) – *n* (%)	8 (31)	9 (56)	3 (27)	1 (33)	21 (37)
Bisphosphonate (Yes) – *n* (%)	11 (42)	16 (100)	11 (100)	3 (100)	41 (73)
Wheelchair use (Yes) – *n* (%)	1 (4) ^**a**^	14 (88) ^**b**^	4 (36) ^**c**^	2 (67)	21 (38)
Oral condition					
DI (Yes) – *n* (%)	5 (19) ^**a**^	11 (69)	6 (55) ^**c**^	0 (0)	22 (39)
Molar Malocclusion Classification – *n* (%)					
Cl I	11 (42)	3 (19)	1 (9)	0 (0)	15 (27)
Cl III	11 (42)	12 (75)	7 (64)	2 (67)	32 (57)
Cl II & mutilated	4 (16)	1 (6)	3 (27)	1 (33)	9 (16)

Statistical tests determine the significant relationship between categorical variables and OI types I, III and IV: Chi-square test or the Fisher’s exact test for contingency tables with small cell counts; Compare means of a continuous variable between OI types I, III and IV: Welch’s t-test for independent samples. As the sample size is small in each group (*n* < 15), results have been confirmed by Mann-Whitney U test (non-parametric test)

^**a**^*p <* 0.05 OI type I compared to OI type III

^**b**^*p <* 0.05 OI type III compared to OI type IV

^**c**^*p <* 0.05 OI type IV compared to OI type I

In children aged 8 to 10 years (Table 1), more children with OI types I and IV were having a parent or ancestor living with OI compared to OI type III (*p*-value < 0.05). All children with OI types III and IV had a history of bisphosphonate treatment (oral or IV) compared with 42% OI type I. Commuting via a wheelchair, an indicator of physical limitaion, was more prevalent in OI type III compared to type IV and in OI type IV more than type I (*p*-value < 0.05). Having DI is more prevalent in OI type III compared to type I (p-value < 0.05). In the group of children (aged 8–10 years), there were no statistical differences in total scores of the CPQ8–10 or domain scores when different types of OI were compared (Table 2).

**Table 2:** The Child Perceptions Questionnaire subscales for 8 to 10-year-old children (CPQ8–10)

OHRQoL	Number of items	Possible range	Observed range	OI I (*n* = 26)	OI III (*n* = 16)	OI IV (*n* = 11)	Others (*n* = 3)	Total (*n* = 56)
Overall	25	0–100	0–43	10.0 (10.5)	9.8 (6.4)	9.0 (7.3)	9 (6.2)	9.7 (8.5)
Oral symptoms	5	0–20	0–15	4.9 (3.8)	4.8 (2.6)	4.4 (1.5)	5.3 (3.1)	4.8 (3.1)
Functional Limitation	5	0–20	0–8	1.3 (1.9)	2.6 (2.5)	1.4 (2.2)	2.0 (1.7)	1.7 (2.2)
Emotional Well-Being	5	0–20	0–20	1.9 (4.2)	1.7 (2.3)	1.7 (3.8)	0.6 (1.2)	1.8 (3.5)
Social Well-Being	10	0–40	0–13	1.8 (3.3)	0.7 (1.3)	1.5 (2.7)	1.0 (1.0)	1.4 (2.7)

Results are shown as n or mean (SD)

***Statistical analysis:*** Welch’s t-test, results have been confirmed by Mann-Whitney U test (non-parametric test)

In adolescents aged 11 to 14 years (Table 3), more teenagers with OI types I were having a parent living with OI compared with OI type III (*p*-value < 0.05). Having chronic pain throughout the body was more prominent in OI type III compared to OI type I (*p*-value < 0.05). All individuals with OI type III and most with OI type IV had a history of bisphosphonate treatment (oral or IV), compared with 51% in OI type I. Using wheelchair as a mean of transportation was more prevalent in OI type III compared to type IV and in OI type IV compared to type I (p-value < 0.05). Having DI is more prevalent in OI type III or IV compared to type I (p-value < 0.05). There were more patients with class III malocclusion amongst teenagers having OI type III compared to type IV and in type IV compared to type I (p-value < 0.05). Total scores of the CPQ11–14 were significantly higher (worse) in OI types III or IV compared to type I (p-value < 0.05 for both). When the sub-scales were compared, functional limitations had a greater negative impact on the OHRQoL of adolescents suffering from OI type III or IV (p-value < 0.05 for both) when compared to those suffering from OI type I (Table 4).

**Table 3:** Characteristics of the 11 to 14-year-old group

Patients aged between 10 and 14	OI I	OI III	OI IV	Others	All
Sociodemographic Characteristics					
Enrolment number – *n* (%)	39 (48)	14 (17)	23 (28)	6 (7)	82 (100)
Female	22 (56)	11 (79)	14 (61)	4 (67)	51 (62)
Age – mean (SD)	13.2 (1.3)	13.4 (1.1)	13.1 (1.2)	13.7 (1.2)	13.2 (1.2)
Race (White) – *n* (%)	32 (82)	12 (86)	19 (83)	4 (67)	67 (82)
others	7 (18)	2 (14)	4 (17)	2 (33)	15 (18)
Insurance status (Private) – *n* (%)	26 (67)	9 (64)	14 (61)	3 (50)	52 (63)
Medicare/Medicaid	13 (33)	5 (36)	9 (39)	3 (50)	30 (37)
Pertinent Medical and Physical Conditions					
Family history (Yes) – *n* (%)	23 (59) ^**a**^	1 (7)	7 (30)	3 (50)	34 (41)
Chronic pain in body (Yes) – *n* (%)	11 (28) ^**a**^	10 (71)	9 (39)	4 (67)	34 (41)
Bisphosphonate (Yes) – *n* (%)	20 (51)	14 (100)	21 (91)	4 (67)	59 (72)
Wheelchair use (Yes) – *n* (%)	1 (3) ^**a**^	13 (93) ^**b**^	10 (43) ^**c**^	4 (67)	28 (34)
Oral conditions					
DI (Yes) – *n* (%)	4 (10) ^**a**^	8 (57)	11 (48) ^**c**^	2 (33)	25 (30)
Molar Malocclusion Classification – *n* (%)					
Cl I	22 (56)	0 (0)	5 (22)	3 (50)	30 (37)
Cl III	8 (20) ^**a**^	14 (100) ^**b**^	13 (56) ^**c**^	2 (33)	37 (45)
Cl II & mutilated	9 (23)	0 (0)	5 (22)	1 (17)	15 (18)

Statistical tests determine the significant relationship between categorical variables and OI types I, III and IV: Chi-square test or the Fisher’s exact test for contingency tables with small cell counts; Compare means of a continuous variable between OI types I, III and IV: Welch’s t-test for independent samples. As the sample size is small in each group (n < 15), results have been confirmed by Mann-Whitney U test (non-parametric test)

^**a**^*p <* 0.05 OI type I compared to OI types III

^**b**^*p <* 0.05 OI type III compared to OI types IV

^**c**^*p <* 0.05 OI type IV compared to OI types I

**Table 4:** The Child Perceptions Questionnaire subscales for 11 to 14-year-old children (CPQ11–14)

OHRQoL	Number of items	Possible range	Observed range	OI I (*n* = 39)	OI III (*n* = 14)	OI IV (*n* = 23)	Others (n = 6)	Total (*n* = 82)
Overall	37	0–148	1–53	16.5 (12.8) ^**a**^	24.7 (12.5)	23.1 (14.4) ^**c**^	22.3 (17.7)	20.2 (13.8)
Oral symptoms	6	0–24	1–11	5.8 (2.9)	7.1 (3.2)	7.1 (3.2)	6.7 (4.3)	6.4 (3.1)
Functional Limitation	9	0–36	0–19	4.3 (4.2) ^**a**^	8.6 (5.1)	7.2 (4.9) ^**c**^	7.4 (5.9)	6.1 (4.9)
Emotional Well-Being	9	0–36	0–20	3.6 (5.7)	5.7 (5.9)	5.5 (6.9)	5.3 (7.2)	4.6 (6.2)
Social Well-Being	13	0–52	0–19	2.9 (4.7)	3.3 (3.8)	3.5 (4.5)	3.0 (3.1)	3.1 (4.3)

Results are shown as n or mean (SD)

Statistical analysis: Welch’s t-test, results have been confirmed by Mann-Whitney U test (non-parametric test)

^**a**^*p <* 0.05 OI type I compared to OI types III

^**b**^*p <* 0.05 OI type III compared to OI types IV

^**c**^*p <* 0.05 OI type IV compared to OI types I

Tables 5 and 6 show the results of multivariable-adjusted ordinal logistic regression for children and teenagers (respectively). Among children, a diagnosis with the more severe type of OI (type IV and III, respectively) was not associated with a negative impact on OHRQoL. Although not statistically significant but having OI type III or IV among children were associated (*p* > 0.05) with a higher grade of functional limitations domain compared to type I (Table 5). However, this association was statistically significant amongst teenagers (Table 6). After adjusting for sociodemographic variables and family history of OI, adolescents having OI type III compared to OI type I have 4.6 (95% CI: 1.2–17.4) times higher odds of having a higher (worse) grade of the CPQ11–14 score (*P* < 0.05). This association was predominantly attributed to the strong correlation between OI types and functional limitations domain (subscale of CPQ). Although the total CPQ_11–14_ score for OI type IV was significantly higher than for OI type I in univariate analysis (Table 4), this difference became statistically insignificant after adjusting for the other variables in the model (Table 6). However, the difference between OI types IV and I persisted with regard to functional limitation (P < 0.05). Having OI type III (OR: 7.8; 95% CI: 1.9–31.7) & IV (OR: 3.7; 95% CI: 1.3–10.3) among adolescents were statistically significantly (P < 0.05) associated with a higher (worse) grade of functional limitations domain compared to OI type I (Table 6).

**Table 5:** Adjusted odds ratio of the negative impact (having higher grades) on the OHRQoL in children

	CPQ_8–10_	Oral Symptoms	Functional Limitation	Emotional Well-Being	Social Well-Being
OI (Type I)	1	1	1	1	1
Type III	1.1 (0.2–4.6)	0.9 (0.2–3.8)	1.6 (0.3–8.3)	2.3 (0.5–11.9)	0.9 (0.2–5.4)
Type IV	0.5 (0.1–2.5)	0.5 (0.1–2.4)	0.7 (0.1–3.5)	0.9 (0.2–5.2)	0.5 (0.1–4.1)
Age	1.6 (0.9–3.1)	1.5 (0.8–2.8)	0.9 (0.5–1.8)	1.2 (0.6–2.4)	1.7 (0.8–3.8)
Gender (Male)	1	1	1	1	1
Female	0.7 (0.2–2.3)	0.9 (0.3–2.9)	0.9 (0.3–3.2)	0.8 (0.2–2.9)	0.3 (0.1–1.2)
Race (White)	1	1	1	1	1
Others	0.9 (0.2–3.4)	2.1 (0.5–8.8)	0.6 (0.1–2.8)	1.7 (0.3–7.8)	4.4 (0.8–24.2)
Family history (No)	1	1	1	1	1
Yes	0.4 (0.1–1.5)	0.4 (0.1–1.7)	0.3 (0.1–1.2)	0.9 (0.2–4.6)	0.2 (0.1–1.3)

Results are given as Odds Ratio (95% Confidence Interval)

**Table 6:** Adjusted odds ratio of the negative impact (having higher grades) on the OHRQoL in adolescents

	CPQ_11–14_	Oral Symptoms	Functional Limitation	Emotional Well-Being	Social Well-Being
OI (Type I)	1	1	1	1	1
Type III	4.6 (1.2–17.4) *	1.8 (0.5–6.6)	7.8 (1.9–31.7)*	3.6 (0.9–13.7)	0.9 (0.3–3.6)
Type IV	2.6 (0.9–7.3)	1.7 (0.6–4.6)	3.7 (1.3–10.3) *	2.3 (0.8–6.6)	1.7 (0.7–4.7)
Age (years)	1.1 (0.8–1.6)	1.1 (0.8–1.6)	1.2 (0.8–1.6)	1.1 (0.7–1.6)	0.9 (0.6–1.3)
Gender (Male)	1	1	1	1	1
Female	0.9 (0.4–2.5)	0.8 (0.3–2.1)	0.7 (0.3–1.9)	0.8 (0.3–2.1)	1.6 (0.7–3.9)
Race (White)	1	1	1	1	1
Others	2.3 (0.6–8.3)	1.8 (0.6–6.3)	1.2 (0.4–4.2)	2.44 (0.7–8.9)	1.4 (0.4–4.5)
Family history (No)	1	1	1	1	1
Yes	1.1 (0.4–2.6)	1.1 (0.4–2.9)	0.9 (0.4–2.6)	1.3 (0.5–3.4)	0.9 (0.3–2.2)

Results are given as Odds Ratio (95% Confidence Interval)

*Statistically Significant findings at *p <* 0.05

## Discussion

In this study we found that adolescents with OI type III had a more negative overall profile of OHRQoL when compared to OI type I. Functional limitation seems to negatively affect OHRQoL of OI types III and IV in comparison with type I amongst teenagers. While sharing similar levels of OHRQoL in three domains (oral symptoms, emotional wellbeing, and social wellbeing), teens with moderate and severe OI (types IV and III) reported worse functional OHRQoL compared to mild OI (type I). This result shows that although the functional limitations in moderate and severe OI affects their perception of physical (functional) oral health QoL, it does not influence their perception of mental and psychological oral health QoL when compared with mild OI.

OI patients’ OHRQoL has not been widely assessed in literature using any of the qualitative questionnaires. Our findings on OHRQoL follows the same pattern as the observation in a study evaluating HRQoL amongst children with different OI severity living in Argentina employing the self-report PedsQL 4.0 questionnaire [10]. Comparing HRQoL of children and adolescents across OI severity groups revealed that physical QoL scores were significantly lower (worse) for children with OI types III and IV with mean PedsQL score of 48.7 compared to those with type I with mean PedsQL score of 66.75 while sharing similar scores for their emotional, school, and social QoL domains [10]. The similarity between the patterns reiterates the fact that the functional limitation is the main perceptional concern when evaluating OHRQoL and HRQoL in children with OI.

This study also describes oral findings in 138 children and teenagers with OI. Similar to our study, prevalence of DI in literature [6–8] is highest in individuals with OI type III ranging from 43 to 100% and in our study occurred in 64% (19 of 30 individuals) of OI type III. Furthermore, the highest occurrence of Class III malocclusion in our study was in OI type III patients with 86% (26 of 30 individuals), earlier reports suggest from 82 to 100% for the same subtype [6–8].

In general, our results in children and teenagers with OI show a better OHRQoL than what has been observed in populations of the same age with a variety of conditions [12, 13, 23]. In children with common dental disease (caries) and orofacial conditions (lip and palate cleft) mean CPQ8–10 scores of 19.1 and 18.4 were found [12], compared to a mean CPQ8–10 score of 9.7 in the present study. This suggests a better OHRQoL amongst children with OI.

Among 11 to 14-year-old adolescents, we found mean CPQ11–14 scores of 24.7 and 23.1 for OI types III and IV, respectively, which is similar to what has been reported for otherwise healthy adolescents with orthodontic disorders and dental caries but better than in adolescents with orofacial conditions (mean CPQ11–14 score: 31.4) [13]. Adolescents with OI type I had significantly lower CPQ11–14 scores compared to the other OI groups or the cohorts reported in the literature.

One interesting observation of the present study was that OHRQoL did not vary between OI types in 8 to 10 year-old children, but was significantly worse in 11 to 14 year-old adolescents with severe OI than in adolescents with mild OI. One explanation for this discrepancy between age groups is that the adolescents experienced functional problems over a longer period of time or perhaps are more conscious of the difficulties caused by the disease.

**Fig. 1:**
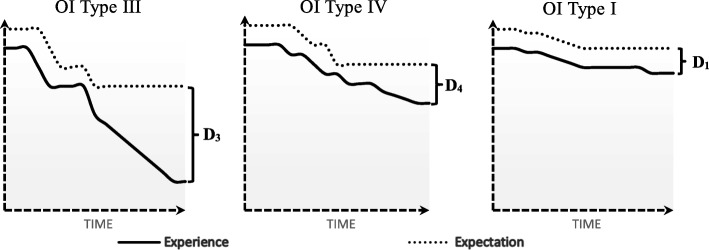
Schematic diagram of the differences in expectation-experience between OI types (D3 > D1; *p* < 0.05)

Quality of life is not static; it is a complex, multifaceted, and dynamic construct that can be defined and measured. It varies between individuals and it changes within the same individual overtime. Consequently, the results of any QoL evaluation has an inherent instability. The relation between oral health status (symptoms) and QoL is not simple nor is it a direct relationship. “People assess their HRQoL by comparing their expectations and experiences” [23]. Expectations are altered and learned by and from experiences. Questionnaires used to evaluate OHRQoL measures the gap between the expectations (hopes) of the individual, and it’s present experience. Existing measures of OHRQoL do not account for expectations of oral health; they only detect the negative impact of the disease or treatment on patient’s perception of oral health QoL. Moreover, patients may be at different points of their illness trajectory when their quality of life is measured [23, 24]. With this understanding, one can interpret the results of this study that the discrepancy (gap) between expectation level and individuals experience is significantly higher in OI types III compare to OI type I at the specific time that their OHRQoL data has been collected with functional limitation being the main contributor. This difference may be due to higher level of expectations or more deteriorating experiences (severe symptoms) or a combination thereof in OI types III compared to those conflicted with OI type I. Therefore, types III are more vulnerable to have lower score in OHRQoL compared to OI type I. (Fig. 1).

Physicians, dentists, and caregivers, in an attempt to provide good care, are trying to bridge the gap between the patients’ experiences and their expectations. In other words, how patients want to be and what their physical health allows them to be. This is commonly achieved through clinical interventions to restore impairments (improve experience) or administering psychological interventions to help them adjust their expectations to their altered clinical health status (diminish expectation).

The OHRQoL scores obtained in this study employing CPQ questionnaire show that these instruments are likely to have “floor effects”, which signifies that they cannot demonstrate improvement in their postinterventional condition. Therefore, given the unique dental issues in OI, it may be useful to develop a more tailored measure for assessing OHRQoL for this population. Disease-specific instruments are generally more sensitive to oral health traits of this condition and more responsive to changes during the time (less “floor effect”) in comparison with generic measures [25, 26].

The main limitation of this study was that there were minimal data on the socioeconomic characteristics of the enrolled participants. Future studies with more comprehensive assessment of patient’s socioeconomic status would complement our data and would offer a better understanding of the relationship between the severity of OI and OHRQoL.

## Conclusions

In conclusion, this study found that teens with OI type III and IV have higher grades of functional limitations than OI type I. This association leads to a lower OHRQoL in teens with OI type III compared with OI type I.

## Additional files

Additional file 1: This file contains Child Perception Questionnaire for children between 8 and 10 years of age. (PDF 305 kb)

Additional file 2: This file contains Child Perception Questionnaire for children between 11 and 14 years of age. (PDF 315 kb)

## Abbreviations

CPQ: Child Perceptions Questionnaire
DI: Dentinogenesis Imperfecta
HRQoL: Health-Related Quality of Life
OHRQoL: Oral Health-Related Quality of Life
OI: Osteogenesis Imperfecta
QoL: Quality of Life
WHO: World Health Organization
