# Supplementary material for: Oral health-related quality of life in children and adolescents with osteogenesis imperfecta: cross-sectional study
Source: Orphanet J Rare Dis. 2018 Oct 25;13:187. doi: 10.1186/s13023-018-0935-y (PMC6202869; doi:10.1186/s13023-018-0935-y)
Supplement: Supplementary file 1 — This file contains Child Perception Questionnaire for children between 8 and 10 years of age. (PDF 305 kb) [file 13023_2018_935_MOESM1_ESM.pdf]

ORAL HEALTH RELATED QUALITY OF LIFE QUESTIONNAIRE
8-10 years

We are doing this study to understand better things that may happen to children because of their teeth and mouth.

.

PLEASE REMEMBER:

- Don't write your name on the questionnaire.
- This is not a test and there are no right or wrong answers.
- Answer as honestly as you can.
- Don't talk to anyone about the questions when you are answering them.
- No one you know will see your answers.
- Read each question carefully and think about the things that have happened to you in the past 4 weeks.
- Before you answer, ask yourself: "Does this happen to me because of my teeth or mouth?"
- Put an x in the box for the answer that is best for you.

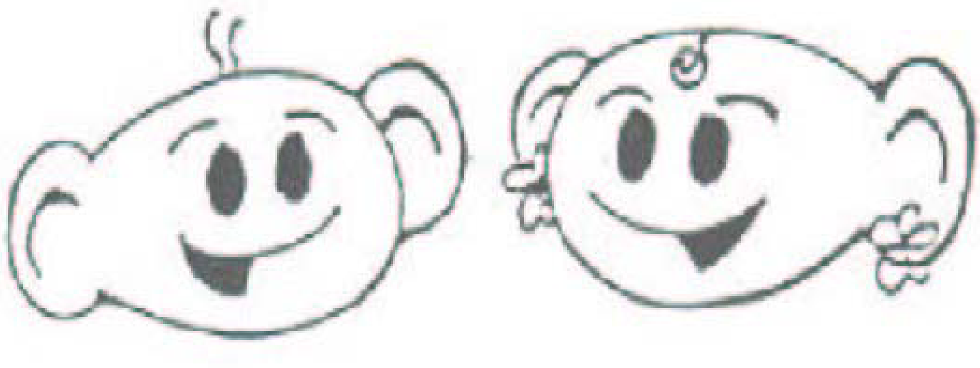

Today's date: ______/______/______
DAY MONTH YEAR

FIRST, A FEW QUESTIONS ABOUT YOU

1. When you think about your teeth or mouth, would you say that they are:
- ❑ Very good
- ❑ Good
- ❑ O.K
- ❑ Poor

2. How much do your teeth or mouth bother you in your everyday life?
- ❑ Not at all
- ❑ A little bit
- ❑ Some
- ❑ A lot

NOW A FEW QUESTIONS ABOUT YOUR TEETH AND MOUTH

How often have you had:

3. Pain in your teeth or mouth in the past four weeks?
- ❑ Never
- ❑ Once or twice
- ❑ Sometimes
- ❑ Often
- ❑ Everyday or almost everyday

4. Sore spots in your mouth in the past four weeks?
- ❑ Never
- ❑ Once or twice
- ❑ Sometimes
- ❑ Often
- ❑ Everyday or almost everyday

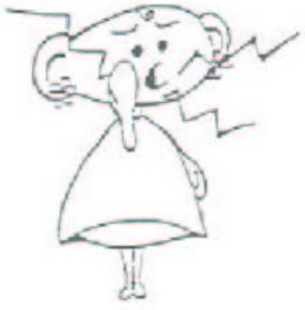

5. Pain in your teeth when you drink cold drinks or eat hot foods in the past 4 weeks?

- ❑ Never
- ❑ Once or twice
- ❑ Sometimes
- ❑ Often
- ❑ Everyday or almost everyday

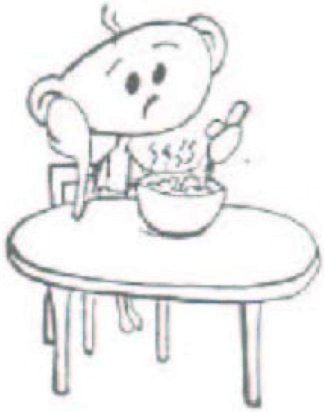

6. Food stuck in your teeth in the past four weeks?

- ❑ Never
- ❑ Once or twice
- ❑ Sometimes
- ❑ Often
- ❑ Everyday or almost everyday

7. Bad breath in the past four weeks?

- ❑ Never
- ❑ Once or twice
- ❑ Sometimes
- ❑ Often
- ❑ Everyday or almost everyday

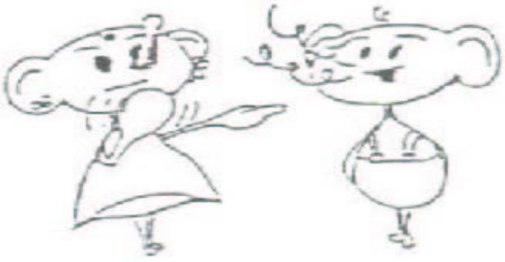

How often have you:

8. Needed longer time than others to eat your meal because of your teeth or mouth in the past 4 weeks?

- ❑ Never
- ❑ Once or twice
- ❑ Sometimes
- ❑ Often
- ❑ Everyday or almost everyday

9. Had a hard time biting or chewing food like apples, corn on the cob or steak because of your teeth or mouth in the past 4 weeks?

- ❑ Never
- ❑ Once or twice
- ❑ Sometimes
- ❑ Often
- ❑ Everyday or almost everyday

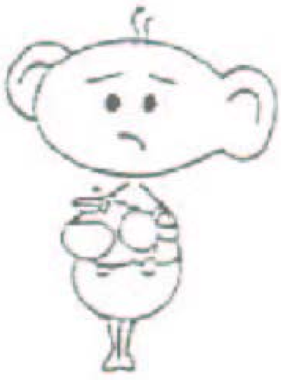

10. Had trouble eating foods you would like to eat because of your teeth or mouth in the past 4 weeks?

- ❑ Never
- ❑ Once or twice
- ❑ Sometimes
- ❑ Often
- ❑ Everyday or almost everyday

11. Had trouble saying some words because of your teeth or mouth in the past 4 weeks?
- ❑ Never
- ❑ Once or twice
- ❑ Sometimes
- ❑ Often
- ❑ Everyday or almost everyday

12. Had a problem sleeping at night because of your teeth or mouth in the past 4 weeks?
- ❑ Never
- ❑ Once or twice
- ❑ Sometimes
- ❑ Often
- ❑ Everyday or almost everyday

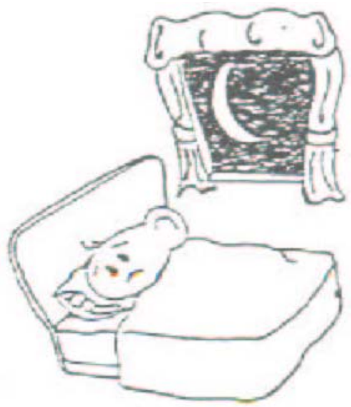

QUESTIONS ABOUT FEELINGS

How often have you:

13. Been upset because of your teeth or mouth in the past 4 weeks?
- ❑ Never
- ❑ Once or twice
- ❑ Sometimes
- ❑ Often
- ❑ Everyday or almost everyday

14. Felt frustrated because of your teeth or mouth in the past 4 weeks?
- ❑ Never
- ❑ Once or twice
- ❑ Sometimes
- ❑ Often
- ❑ Everyday or almost everyday

15. Been shy because of your teeth or mouth in the past four weeks?
- ❑ Never
- ❑ Once or twice
- ❑ Sometimes
- ❑ Often
- ❑ Everyday or almost everyday

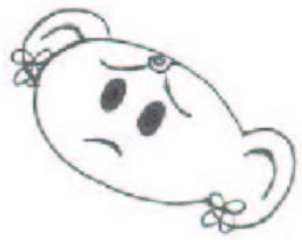

16. Been concerned what other people think about your teeth or mouth in the past four weeks?
- ❑ Never
- ❑ Once or twice
- ❑ Sometimes
- ❑ Often
- ❑ Everyday or almost everyday

17. Worried that you are not as good-looking as others because of your teeth or mouth in the past four weeks?
- ❑ Never
- ❑ Once or twice
- ❑ Sometimes
- ❑ Often
- ❑ Everyday or almost everyday

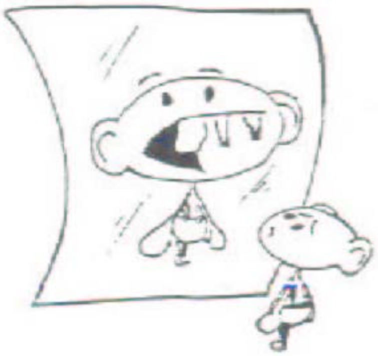

QUESTIONS ABOUT YOUR SCHOOL

How often have you:

18. Missed school because of your teeth or mouth in the past four weeks?

- ❑ Never
- ❑ Once or twice
- ❑ Sometimes
- ❑ Often
- ❑ Everyday or almost everyday

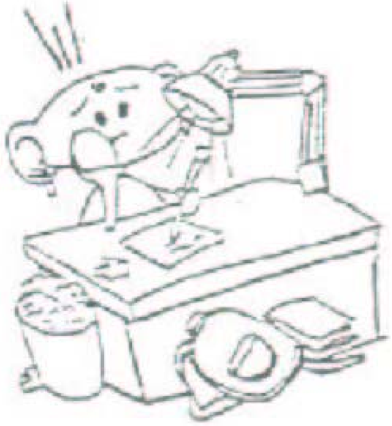

19. Had a hard time doing your homework because of your teeth or mouth in the past four weeks?
- ❑ Never
- ❑ Once or twice
- ❑ Sometimes
- ❑ Often
- ❑ Everyday or almost everyday

20. Had a hard time paying attention in school because of your teeth or mouth in the past 4 weeks?

- ❑ Never
- ❑ Once or twice
- ❑ Sometimes
- ❑ Often
- ❑ Everyday or almost everyday

21. Not wanted to speak or read out loud in class because of your teeth or mouth in the past 4 weeks?

- ❑ Never
- ❑ Once or twice
- ❑ Sometimes
- ❑ Often
- ❑ Everyday or almost everyday

QUESTIONS ABOUT YOU BEING WITH OTHER PEOPLE

How often have you:

22. Stayed away from activities like sports and clubs because of your teeth or mouth in the past four weeks?

- ❑ Never
- ❑ Once or twice
- ❑ Sometimes
- ❑ Often
- ❑ Everyday or almost everyday

23. Not wanted to talk to other children because of your teeth or mouth in the past four weeks?

- ❑ Never
- ❑ Once or twice
- ❑ Sometimes
- ❑ Often
- ❑ Everyday or almost everyday

24. Tried not to smile or laugh when with other children because of your teeth or mouth in the past four weeks?

- ❑ Never
- ❑ Once or twice
- ❑ Sometimes
- ❑ Often
- ❑ Everyday or almost everyday

25. Not wanted to be with other children because of your teeth or mouth in the past four weeks?

- ❑ Never
- ❑ Once or twice
- ❑ Sometimes
- ❑ Often
- ❑ Everyday or almost everyday

How often have:

26. Other children teased you or called you names because of your teeth or mouth in the past 4 weeks?

- ❑ Never
- ❑ Once or twice
- ❑ Sometimes
- ❑ Often
- ❑ Everyday or almost everyday

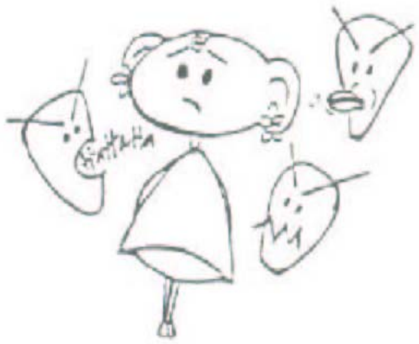

27. Other children asked you questions about your teeth or mouth in the past four weeks?

- ❑ Never
- ❑ Once or twice
- ❑ Sometimes
- ❑ Often
- ❑ Everyday or almost everyday

THERE, IT'S FINISHED!

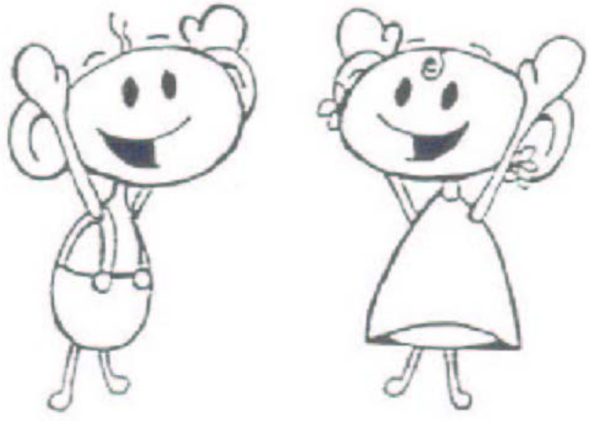

THANK YOU FOR HELPING US
