# Supplementary material for: Oral health-related quality of life in children and adolescents with osteogenesis imperfecta: cross-sectional study
Source: Orphanet J Rare Dis. 2018 Oct 25;13:187. doi: 10.1186/s13023-018-0935-y (PMC6202869; doi:10.1186/s13023-018-0935-y)
Supplement: Supplementary file 2 — This file contains Child Perception Questionnaire for children between 11 and 14 years of age. (PDF 315 kb) [file 13023_2018_935_MOESM2_ESM.pdf]

ORAL HEALTH RELATED QUALITY OF LIFE QUESTIONNAIRE
11-18 years

This questionnaire will help us better understand problems children may have with their teeth, mouth, lips and jaws. By answering the questions, you will help us learn more about young people's experiences.

PLEASE REMEMBER:

- Don't write your name on the questionnaire.
- This is not a test and there are no right or wrong answers.
- Answer as honestly as you can. Don't talk to anyone about the questions when you are answering them. Your answers are private; no one you know will see them.
- Read each question carefully and think about your experiences in the past 3 months when you answer.
- Before you answer, ask yourself: "Does this happen to me because of problems with my teeth, lips, mouth or jaws?"
- Put an x in the box for the answer that is best for you.

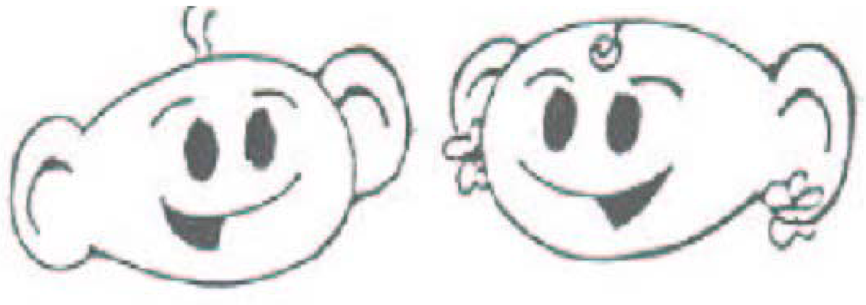

Today's date: ______/______/______
DAY MONTH YEAR

FIRST, A FEW QUESTIONS ABOUT YOU

1. Would you say the health of your teeth, lips, jaws and mouth is:
- ❑ Excellent
- ❑ Very good
- ❑ Good
- ❑ Fair
- ❑ Poor

2. How much does the condition of your teeth, lips, jaws or mouth affect your life overall?
- ❑ Not at all
- ❑ Very little
- ❑ Some
- ❑ A lot
- ❑ Very much

QUESTIONS ABOUT ORAL PROBLEMS

In the past 3 months, how often have you had:

3. Pain in your teeth, lips, jaws or mouth?
- ❑ Never
- ❑ Once or twice
- ❑ Sometimes
- ❑ Often
- ❑ Everyday or almost everyday

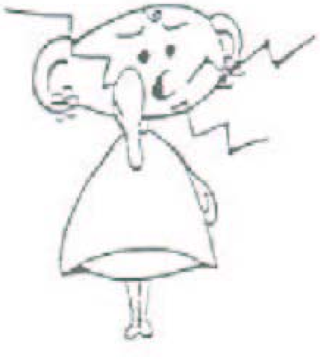

4. Bleeding gums?
- ❑ Never
- ❑ Once or twice
- ❑ Sometimes
- ❑ Often
- ❑ Everyday or almost everyday

5. Sores in your mouth?
❑ Never
❑ Once or twice
❑ Sometimes
❑ Often
❑ Everyday or
almost everyday

6. Bad breath?
❑ Never
❑ Once or twice
❑ Sometimes
❑ Often
❑ Everyday or
almost everyday

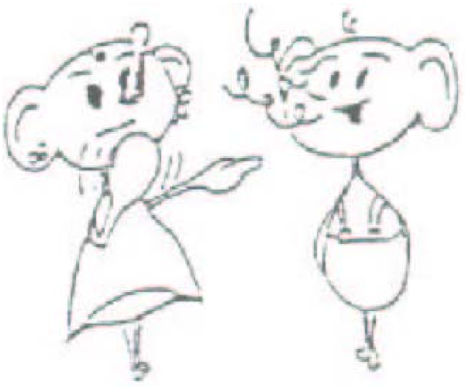

7. Food stuck in or between your teeth?
❑ Never
❑ Once or twice
❑ Sometimes
❑ Often
❑ Everyday or
almost everyday

8. Food stuck in the top of your mouth?
❑ Never
❑ Once or twice
❑ Sometimes
❑ Often
❑ Everyday or
almost everyday

For the next questions…
Has this happened because of your <u>teeth, lips, jaws or mouth?</u>
In the past <u>3 months</u>, how often have you had:

9. Breathed through your mouth?
❑ Never
❑ Once or twice
❑ Sometimes
❑ Often
❑ Everyday or
almost everyday

10. Taken longer than others to eat a meal?
❑ Never
❑ Once or twice
❑ Sometimes
❑ Often
❑ Everyday or
almost everyday

11. Had trouble sleeping?
- ❑ Never
- ❑ Once or twice
- ❑ Sometimes
- ❑ Often
- ❑ Everyday or almost everyday

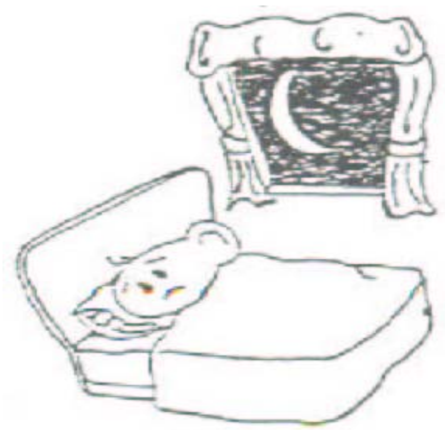

In the past <u>3 months</u>, because of your <u>teeth, lips, mouth or jaws</u>, how often has it been:

12. Difficult to bite or chew food like apples, corn on the cob or steak?
- ❑ Never
- ❑ Once or twice
- ❑ Sometimes
- ❑ Often
- ❑ Everyday or almost everyday

13. Difficult to open your mouth wide?
- ❑ Never
- ❑ Once or twice
- ❑ Sometimes
- ❑ Often
- ❑ Everyday or almost everyday

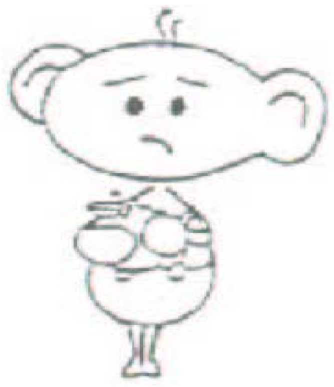

14. Difficult to say any words?
- ❑ Never
- ❑ Once or twice
- ❑ Sometimes
- ❑ Often
- ❑ Everyday or almost everyday

15. Difficult to eat foods you would like to eat?
- ❑ Never
- ❑ Once or twice
- ❑ Sometimes
- ❑ Often
- ❑ Everyday or almost everyday

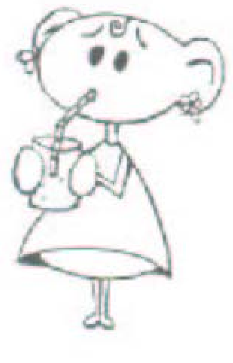

16. Difficult to drink with a straw?
- ❑ Never
- ❑ Once or twice
- ❑ Sometimes
- ❑ Often
- ❑ Everyday or almost everyday

17. Difficult to drink or eat hot or cold foods?

❑ Never
❑ Once or twice
❑ Sometimes
❑ Often
❑ Everyday or almost everyday

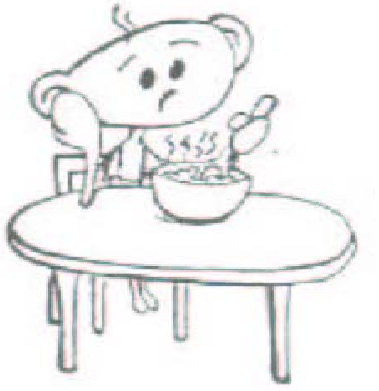

QUESTIONS ABOUT FEELINGS

Have you had the feeling because of your teeth, lips, jaws or mouth?
If you felt this way for another reason, answer 'Never'.

In the past <u>3 months</u>, how often have you:

18. Felt irritable or frustrated?

❑ Never
❑ Once or twice
❑ Sometimes
❑ Often
❑ Everyday or almost everyday

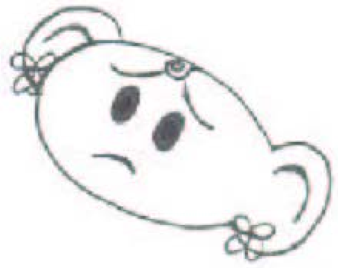

19. Felt unsure of yourself?

❑ Never
❑ Once or twice
❑ Sometimes
❑ Often
❑ Everyday or almost everyday

20. Felt shy or embarrassed?

❑ Never
❑ Once or twice
❑ Sometimes
❑ Often
❑ Everyday or almost everyday

In the past 3 months, because of your teeth, lips, mouth or jaws, how often have you:

21. Been concerned what other people think about your teeth, lips, mouth or jaws?

- ❑ Never
- ❑ Once or twice
- ❑ Sometimes
- ❑ Often
- ❑ Everyday or almost everyday

22. Worried that you are not as good-looking as others?

- ❑ Never
- ❑ Once or twice
- ❑ Sometimes
- ❑ Often
- ❑ Everyday or almost everyday

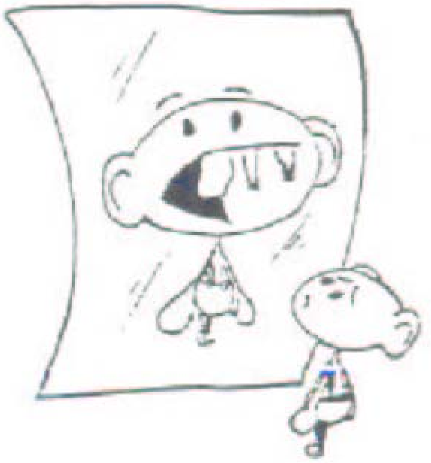

23. Been upset?

- ❑ Never
- ❑ Once or twice
- ❑ Sometimes
- ❑ Often
- ❑ Everyday or almost everyday

24. Felt nervous or afraid?

- ❑ Never
- ❑ Once or twice
- ❑ Sometimes
- ❑ Often
- ❑ Everyday or almost everyday

25. Worried that you are not as healthy as others?

- ❑ Never
- ❑ Once or twice
- ❑ Sometimes
- ❑ Often
- ❑ Everyday or almost everyday

26. Worried that you are different than other people?

- ❑ Never
- ❑ Once or twice
- ❑ Sometimes
- ❑ Often
- ❑ Everyday or almost everyday

QUESTIONS ABOUT SCHOOL

Have you had these experiences because of your <u>teeth, lips, jaws or mouth</u>? If it was for <u>another reason</u>, answer 'Never'.

In the past <u>3 months</u>, how often have you:

27. Missed school because of pain, appointments, or surgery?
❑ Never
❑ Once or twice
❑ Sometimes
❑ Often
❑ Everyday or almost everyday

28. Had a hard time paying attention in school?
❑ Never
❑ Once or twice
❑ Sometimes
❑ Often
❑ Everyday or almost everyday

29. Had difficulty doing your homework?
❑ Never
❑ Once or twice
❑ Sometimes
❑ Often
❑ Everyday or almost everyday

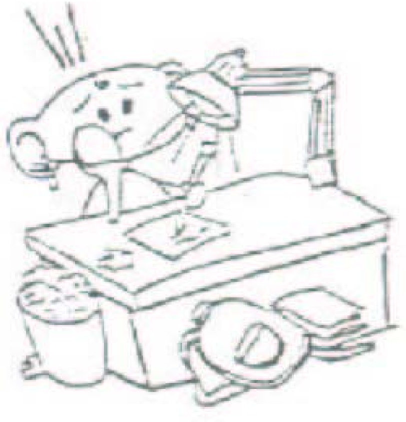

30. Not wanted to speak or read out loud in class?
❑ Never
❑ Once or twice
❑ Sometimes
❑ Often
❑ Everyday or almost everyday

QUESTIONS ABOUT YOUR SPARE-TIME ACTIVITIES & BEING WITH OTHER PEOPLE

Have you had these experiences because of your <u>teeth, lips, jaws or mouth</u>? If it was for <u>another reason</u>, answer 'Never'.

In the past <u>3 months</u>, how often have you:

31. Avoided taking part in activities like sports, clubs, drama, music, school trips?
❑ Never
❑ Once or twice
❑ Sometimes
❑ Often
❑ Everyday or almost everyday

32. Not wanted to talk to other children?
❑ Never
❑ Once or twice
❑ Sometimes
❑ Often
❑ Everyday or almost everyday

33. Avoided smiling or laughing when around other children?
❑ Never
❑ Once or twice
❑ Sometimes
❑ Often
❑ Everyday or almost everyday

34. Had difficulty playing a musical instrument such as a recorder, flute, clarinet, trumpet?
❑ Never
❑ Once or twice
❑ Sometimes
❑ Often
❑ Everyday or almost everyday

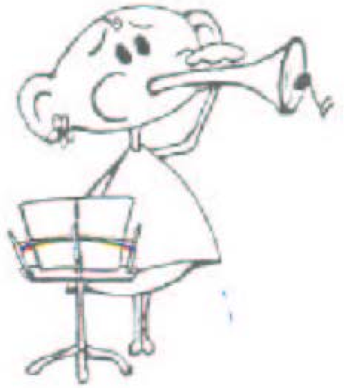

35. Not wanted to spend time with other children?
❑ Never
❑ Once or twice
❑ Sometimes
❑ Often
❑ Everyday or almost everyday

36. Argued with other children or your family?
- ❑ Never
- ❑ Once or twice
- ❑ Sometimes
- ❑ Often
- ❑ Everyday or
almost everyday

In the past <u>3 months</u>, because of your <u>teeth, lips, mouth or jaws</u>, how often have:

37. Other children teased you or called you names?
- ❑ Never
- ❑ Once or twice
- ❑ Sometimes
- ❑ Often
- ❑ Everyday or
almost everyday

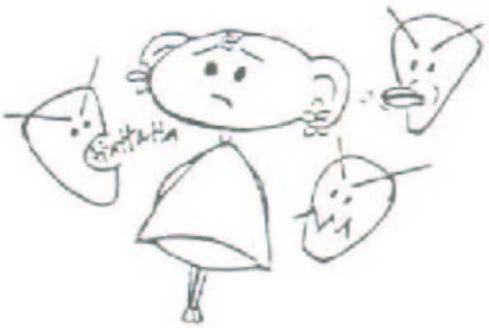

38. Other children made you feel left out?
- ❑ Never
- ❑ Once or twice
- ❑ Sometimes
- ❑ Often
- ❑ Everyday or almost everyday

39. Other children asked you questions about your teeth, lips, jaws or mouth?
- ❑ Never
- ❑ Once or twice
- ❑ Sometimes
- ❑ Often
- ❑ Everyday or
almost everyday

THERE, IT'S FINISHED!

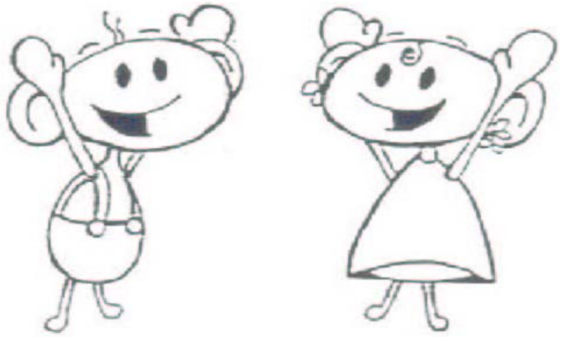

THANK YOU FOR HELPING US
